# Fecal Microbiota Transplantation during and Post-COVID-19 Pandemic

**DOI:** 10.3390/ijms22063004

**Published:** 2021-03-16

**Authors:** Negin Kazemian, Dina Kao, Sepideh Pakpour

**Affiliations:** 1 School of Engineering, University of British Columbia, Kelowna, BC V1V 1V7, Canada; negin.kazemian@ubc.ca; 2 Division of Gastroenterology, Department of Medicine, University of Alberta, Edmonton, AB T6G 2G3, Canada; dkao@ualberta.ca

**Keywords:** fecal microbiota transplantation (FMT), *Clostridioides difficile* infection (CDI), gut microbiome, COVID-19, policy guidelines

## Abstract

COVID-19 is a major pandemic facing the world today, which has implications on current microbiome-based treatments such as fecal microbiota transplantation (FMT) used for recurrent *Clostridioides difficile* infections. The bidirectional relationship between the inhabitants of our gut, the gut microbiota, and COVID-19 pathogenesis, as well as the underlying mechanism involved, must be elucidated in order to increase FMT safety and efficacy. In this perspective, we discuss the crucial cross-talk between the gut microbiota and the lungs, known as the gut–lung axis, during COVID-19 infection, as well as the putative effect of these microorganisms and their functional activity (i.e., short chain fatty acids and bile acids) on FMT treatment. In addition, we highlight the urgent need to investigate the possible impact of COVID-19 on FMT safety and efficacy, as well as instilling stringent screening protocols of donors and recipients during COVID-19 and post-COVID-19 pandemic to produce a cohesive and optimized FMT treatment plan across all centers and in all countries across the globe.

## 1. Introduction

Fecal microbiota transplantation (FMT) is a novel treatment that is highly effective in the management of recurrent *Clostridioides difficile* infections (rCDI), and holds promise in several dysbiosis associated diseases [1,2,3,4]. FMT is the transfer of distal gut microbial communities from a healthy individual to a patient’s intestinal tract [5]. This treatment has been shown to restore a disturbed microbial ecosystem and related microbial functional networks, leading to a ~90% cure rate for rCDI patients [4]. The logistics of selecting and screening donors as well as optimizing the efficacy of this treatment are the main constraints of the therapeutic uses of FMT [6]. Past surveys have shown that 50% of physicians have identified the complexity and cost of donor screening as the main barrier in providing FMT [7]. The safety and efficacy concerns are further exacerbated by the current outbreak of coronavirus disease 2019 (COVID-19), caused by severe acute respiratory syndrome coronavirus 2 (SARS-CoV-2) [8]. As of 16 February 2021, over 108,822,960 cases have been confirmed worldwide, with a total death count of ~2,403,641 [9]. In addition, silent spreaders (asymptomatic individuals) exist and can potentially transmit the virus [10,11]. Although no cases of COVID-19 transmitted through FMT treatment have been reported, the potential of such transmission remains unknown. In addition, further research is required to uncover how COVID-19 may potentially affect a donors’ and recipients’ gut microbiome after recovery from COVID-19, and if any long-term effects exist on their microbiome composition and function, which may affect FMT efficacy. Such constraints could pose new challenges for the future of FMT treatment; therefore, it is important to adapt and improve current FMT guidelines and policies during and post-COVID-19 from two different aspects, safety and efficacy.

**Figure 1:**
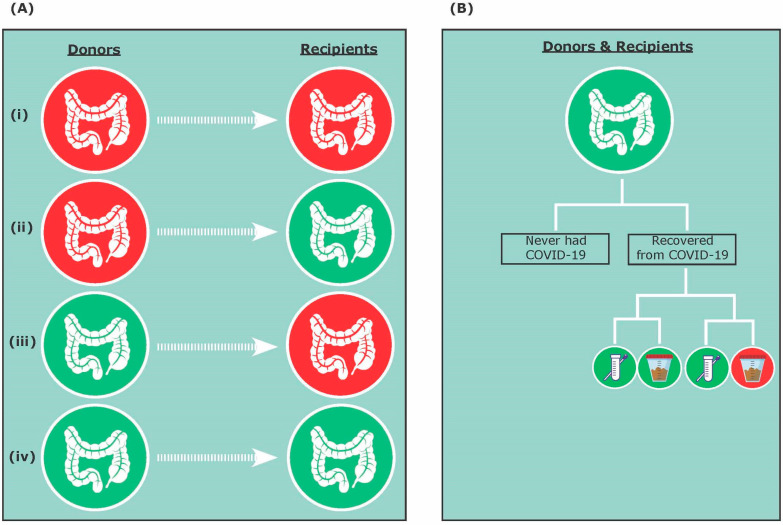
Fecal microbiota transplantation (FMT) treatment during COVID-19 and post-COVID-19 pandemic. (**A**) The four possible FMT donor and recipient combinations include: (**i**) asymptomatic COVID-19 donor and asymptomatic COVID-19 recipient; (**ii**) asymptomatic COVID-19 donor and COVID-19-negative recipient; (**iii**) COVID-19-negative donor and asymptomatic COVID-19 recipient; and (**iv**) COVID-19-negative donor and COVID-19-negative recipient. (**B**) Donors and recipients that have tested negative for COVID-19 could have never had COVID-19 or have recovered from COVID-19. Recovering donors and recipients could have a negative nasopharyngeal test and a negative stool test, or a negative nasopharyngeal test and a positive stool test.

We propose that four possible combinations and scenarios can exist for FMT during COVID-19 and post-COVID-19 pandemic, including (i) asymptomatic COVID-19 donor and asymptomatic COVID-19 recipient; (ii) asymptomatic COVID-19 donor and COVID-19-negative recipient; (iii) COVID-19-negative donor and asymptomatic COVID-19 recipient; and (iv) COVID-19-negative donor and COVID-19-negative recipient (Figure 1A). In addition, donors and recipients that are COVID-19-negative could have either never contracted COVID-19 or have recovered from COVID-19 (Figure 1B). Therefore, this review will discuss FMT efficacy and safety during COVID-19 and post-COVID-19 pandemic considering both donors and recipients, as well as present practical advice for clinicians interested in best practices around the delivery of FMT.

## 2. COVID-19 and FMT Safety

There are several approaches for FMT donor selection, including patient-directed, unrelated, and autologous donors [12,13]. For patient-directed donor selection, a family member or a friend is usually selected to donate to a single recipient [12]. An unrelated donor is typically unknown to the recipient and donates to multiple recipients [12]. Lastly, fecal microbiota from an individual can be banked and administered at a later date [13]. Although highly effective in the treatment of rCDI, the long-term consequences of FMT remain to be determined, because this treatment can carry infectious and non-infectious risks [14,15,16]. Thus, donors should be healthy without known gastrointestinal disease or recent antibiotic therapy (within 90 days). Donors should also be excluded if they have had a history of disease potentially associated with alterations in the gut microbiota (e.g., autoimmune disease, metabolic syndrome, obesity, etc.) [12,17]. In addition, stool banks and centers often conduct intensive screening protocols for FMT donors including serological and stool tests. Serologic tests often screen for several bacteria, viruses, and parasites including Hepatitis A, Hepatitis B, Hepatitis C, Hepatitis E, human immunodeficiency viruses (HIV) ½, Human T-lymphotropic virus (HTLV), and *Treponema pallidum* [13,18]. Safety concerns with the transmission of multi-drug resistant organisms, extended-spectrum beta-lactamase (ESBL)-producing *Escherichia coli* and Shiga-toxin producing *E. coli,* have also led to additional testing requirements [14,19]. In contrast to the general consensus for minimal donor serological testing, there is more uncertainty of how extensive the stool screening tests need to be and can vary significantly between centers and countries [13]. To further improve safety, a quarantine model is proposed where all FMT materials are held between two screening time points and released only after all tests are negative. However, this model does not completely eliminate transmission of infection because an infection may potentially be acquired, or asymptomatic shedding of viruses can occur in between the two screening time points. Therefore, the need to develop more consistent and evidence-based screening protocols for FMT during and after the COVID-19 pandemic is more crucial than ever.

SARS-CoV-2 primarily causes lung infection by utilizing the angiotensin-converting enzyme 2 (ACE2) receptors present on the alveolar epithelial cell [20,21]. ACE2 receptors are also present elsewhere in the body, including the kidney, heart, liver, eyes, the epithelial cells of the oral mucosa, and the gut [22]. Although the transmission of SARS-CoV-2 is thought to occur mainly via respiratory droplets, the gut may also contribute toward the pathogenesis of COVID-19, because the intestinal epithelial cells, particularly the enterocytes of the small intestine, also express ACE2 receptors [23,24,25,26]. Past studies have also reported gastrointestinal symptoms and the presence of SARS-CoV-2 viral particles in stool samples of infected individuals [26,27]. In a study by Xiao and colleagues (2020), some patients continued to have detectable viral RNA in their stool after negative results in their respiratory samples [26]. In addition, it has been shown that ACE2 expression in the gut is downregulated during SARS-CoV-2 infection, leading to reduced secretion of antimicrobial peptides (AMPs) and increased pathogen survival [28,29]. Thus, as the COVID-19 pandemic spreads across the globe, there is an urgent need to take precautions and screen FMT donors for SARS-CoV-2 to prevent the potential risk of COVID-19 transmission. We speculate that COVID-19 may be transmitted via FMT from asymptomatic donors to recipients, specifically from those who may have tested negative for COVID-19 via their respiratory samples but positive in their fecal samples (Figure 1B).

To address this concern, FMT donor screening should be stringent and follow guidelines at each jurisdiction. Presently, the FDA recommends that only FMT products generated from stool donated before 1 December 2019, can be used until proper testing and screening protocols are available [30]. As such, the OpenBiome, a non-profit public stool bank, has been following this guideline and testing the stool of all donors after that date [31]. If the donors have a positive test, all materials will be destroyed 28 days prior to the test date [31]. In addition, the donor will be placed on hold and excluded from providing donations for a minimum of eight weeks [31]. The FMT centre at the Chinese University of Hong Kong, one of the largest providers of FMT in Asia, has also quarantined all donor material donated since 1 November 2019, as a precaution [32]. If stool donated before the recommended FDA timeline is not available, it is vital to screen donors for COVID-19 using stool samples. Ng and colleagues (2020) indicated that a single negative test for stool is insufficient to exclude the presence of SARS-CoV-2 [32]. Thus, multiple testing at different time points may be necessary. Donor consent will need to be amended to accept the potential risk of being infected with COVID-19 during the testing and donation process [30] and to notify the donor program should there be any exposure risks of SARS-CoV-2 or symptoms suggestive of COVID-19. In addition, the initial interview can be conducted virtually to protect the candidate donor as well as healthcare providers [33]. Previously, Ianiro and colleagues discussed that physicians should screen for (i) the presence of typical COVID-19 symptoms within the previous 30 days; and (ii) the donor’s history of travel to regions affected by COVID-19 or close contact with individuals with proven or suspected infection within the previous 30 days [34]. Thus, during the clinical assessment, if the potential donor has any symptoms suggestive of COVID-19, they should be excluded from the next stage of laboratory screening and donation processes. If the donors pass the questionnaire, they will undergo laboratory testing including SARS-CoV-2 [6]. Although testing for SARS-CoV-2 can be performed through a nasopharyngeal swab, direct examination of stool samples is preferred if a validated test is available. In a recent Nature poll, 89% of scientists felt that SARS-CoV-2 was either very likely or likely to become an endemic virus [35]. Thus, Ianiro and colleagues have suggested that in endemic countries, the real-time reverse transcription-polymerase chain reaction (RT-PCR) assay should be considered for all donors [34]. It is also important to mention that prior to COVID-19, a significant proportion of potential donors (60–90%) could fail screening protocols, most of them during the health interview and physical examination [36,37]. We acknowledge that this failure rate may increase during COVID-19 and post-COVID-19, making donor recruitment and retention even more challenging. With new emerging pathogens which can be transmitted through stool, donor screening will need to continuously evolve; however, it still does not completely eliminate the risks of disease transmission. Thus, more refined biotherapeutics are urgently needed. This has led to the exploration of sterile fecal filtrate transfer and its efficacy for rCDI. In a preliminary study by Ott and colleagues, sterile fecal filtrate transfer prevented recurrent rCDI in five patients [38]. SER-109, containing only spore-forming Firmicutes can also be used because the manufacturing process uses ethanol to treat donor stool, thus eliminating all vegetative bacteria [39]. In a phase 2 clinical trial with 89 rCDI patients, early engraftment of SER-109 has been shown to reduce CDI recurrence [39]. Microbial ecosystems therapeutics (MET-2), the first defined and donor-independent biotherapeutic, is an oral encapsulated formulation of 40 lyophilized commensal bacterial species [40]. They were initially isolated from the stool of a healthy donor, but subsequently manufactured independently of stool donors. In a phase 1 open-label trial, MET-2 showed comparable efficacy to FMT preventing CDI recurrence, providing proof of principle for defined biotherapeutics [40].

Moreover, when considering how FMT is to be administered to recipients, a non-invasive route, such as oral capsules, would be preferable, because it will reduce potential exposure risk to healthcare providers, without reducing clinical efficacy. However, FMT oral capsules may not be widely available, and may not be possible for recipients who have dysphagia. Ultimately, the decision to proceed with FMT should be considered on a case-by-case basis. Considerations for postponing the procedures during the COVDI-19 pandemic may also be deliberated, unless there are high-risk patients with fulminant and antibiotic refractory CDI where FMT could be lifesaving.

## 3. COVID-19 and FMT Efficacy

It is known that gut microbiota plays a vital role in human metabolism, immunity, and diseases. To date, the efficacy of FMT has been ascribed to the restoration of a normal gut microbiome composition and function via engraftment, and a sustained coexistence of donor and recipient bacterial strains [41]. Furthermore, the cross-talk between the lungs and the gut microbiota, known as the gut–lung axis, is bidirectional; the gut microbiota can directly (via metabolites) or indirectly (via the immune system) impact the lung, and the distinct microorganisms in the lung and its inflammation can also affect the gut microbiota [42,43]. For example, the gut microbiota is broadly protective against respiratory infections, because its depletion leads to impaired immune responses and worsens outcomes following bacterial or viral respiratory infection [44,45,46]. In a study by Zhang and colleagues, the pathogenic role of gut microbiota was also highlighted in common lung diseases including asthma, chronic obstructive pulmonary disease (COPD), respiratory infections, cystic fibrosis, and lung cancer [47]. In addition, viral lung infections can alter the gut microbiota [48]. The gut–lung axis is only beginning to be understood, and our understanding of these interactions remains in its infancy.

**Figure 2:**
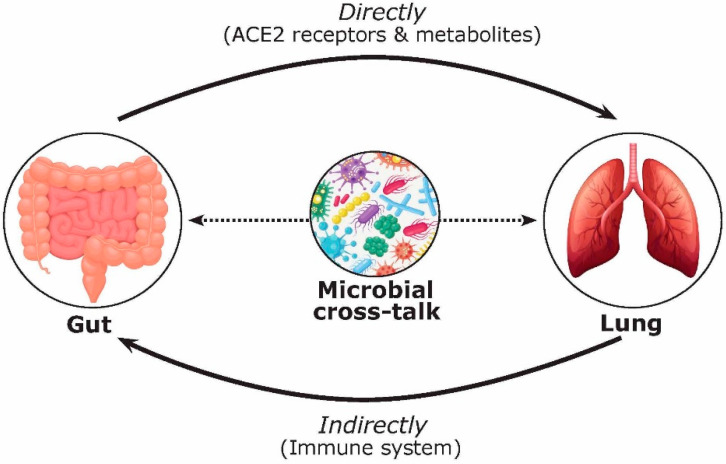
Multifaceted mechanisms involved in the gut–lung axis during COVID-19 infection affecting FMT safety and efficacy. The bidirectional effects of the gut–lung axis are complex and can take place via direct (i.e., ACE2 receptors and metabolites including short chain fatty acids (SCFAs) and bile acids) and indirect (i.e., immune system) mechanisms.

The gut–lung axis has generated a significant amount of coverage since the start of the pandemic [42,43,47,49]. For example, a review by de Oliveira and colleagues (2021) highlighted the gut–lung axis and its dysbiosis, as well as the common gastrointestinal manifestations in COVID-19 [49]. However, the discussion of the functional activity and metabolites of microorganisms in this cross-talk, as well as their effect on FMT treatment, has been overlooked. Focusing on COVID-19, we hypothesize that the bidirectional gut–lung axis during COVID-19 infection can directly (via ACE2 receptors and gut microbial metabolites such as short chain fatty acids (SCFAs) and bile acids) and indirectly (via the immune system) affect the gut and lung (Figure 2). Previous studies have shown that over 60% of patients with COVID-19 have gastrointestinal manifestations and reported evidence of gastrointestinal symptoms such as diarrhea, nausea, and vomiting [50,51]. Moreover, several reports have pointed to alternations in the gut microbiota composition in COVID-19 patients [29,52,53,54,55]. For example, in a study by Zuo and colleagues (2020), twenty-three bacterial taxa showed a significant positive correlation with COVID-19 disease severity [52]. Positive associations were shown between COVID-19 disease severity and bacterial members from the phylum Firmicutes, the genus *Coprobacillus*, as well as species *Clostridium ramosum* and *Clostridium hathewayi* [23]. They also described several *Bacteroides* species (*B. dorei*, *B. thetaiotamicron*, *B. massiliensis*, and *B. ovatus*) that were inversely correlated with viral load, which could interestingly downregulate the ACE2 receptor in the murine gut [52]. Patients with COVID-19 have also had a higher abundance of opportunistic pathogens such as *Collinsella aerofaciens*, *Collinsella tanakaei*, *Streptococcus infantis*, *Morganella morganii*, and *Bifidobacterium dentium* compared to healthy participants [54,55]. Moreover, opportunistic fungi were also observed in COVID-19 patients including *Aspergillus* and *Candida* spp. [52,56]. In contrast, beneficial commensals such as *Bifidobacterium adolescentis*, *Eubacterium rectale*, *Blautia obeum*, *Dorea formicigenerans*, *Alistipes onderdonkii*, and *Faecalibacterium prausnitzii* were negatively associated with COVID-19 severity [52,55]. This is intriguing because *F. prausnitzii* is a key synthesizer of SCFAs [57]. SCFAs are formed due to the fermentation of complex carbohydrates affecting a range of host processes including host–microbe signalling, energy utilization, and the control of colonic pH with consequent effects on the microbiota composition and gut motility [58]. The most abundant SCFAs are acetate, propionate, and butyrate [59]. These microbial-derived metabolites also have anti-inflammatory effects within the gut and in the airways, which may explain their protective effect against COVID-19 infection [57,60]. Hence, the role of biotherapeutics could represent an important tool for the control of excessive inflammation in COVID-19 and reduce the incidence, duration, and severity of viral respiratory infections [49].

Other microbial metabolites such as bile acids may also affect COVID-19 pathogenesis and FMT efficacy. Primary bile acids are conjugated to the amino acids taurine or glycine to form bile salts, which are secreted and stored in the gallbladder until they are released into the small intestine [61]. In the gut, primary bile acids such as cholic acid (CA) and chenodeoxycholic acid (CDCA) become conjugated by the gut microbiota and bile salt hydrolase (BSH) to form secondary bile acids, including deoxycholic acid (DCA), lithocholic acid (LCA), and ursodeoxycholic acid (UDCA) [61,62]. Previous studies have shown that stool samples from rCDI patients were enriched with taurocholic acid, a potent *C. difficile* germinant, and show decreases in LCA and DCA, secondary bile acids that inhibit *C. difficile* germination prior to FMT [63,64]. Interestingly, emerging evidence suggests that CDCA and secondary bile acid (tauroursodeoxycholic acid) may inhibit rotavirus, hepatitis B/D, and Influenza A, as well as Influenza A and hepatitis B virus infection, respectively [65,66,67,68]. Bile acids have been proposed to possess anti-inflammatory properties and can inhibit the NF-κB-dependent transcription of pro-inflammatory cytokines via farnesoid X receptor (FXR) and membrane G protein-coupled bile acid receptor Gpbar-1 (also known as TGR5) [69]. UDCA, for example, has been shown to inhibit pro-inflammatory cytokines such as TNF-α, IL-1β, IL-2, IL-4, and IL-6 [70,71]. In addition, UDCA has also been shown to stimulate alveolar fluid clearance in lipopolysaccharide-induced pulmonary edema, resulting in an improvement of acute respiratory distress syndrome [72]. Thus, the potential protective and therapeutic role of bile acids must be further explored in COVID-19 infection.

Increased proinflammatory cytokines, known as the “cytokine storm” is associated with severe SARS-CoV-2 infection [73,74] and clearly reflects an uncontrolled dysregulation of the host’s immune function. This enhanced cytokine and chemokine production can lead to severe acute respiratory syndrome in the lungs and multiple organ failure [73,75]. The gut microbiota not only affects the innate immune response but also boosts CD8+ T cell effector function, which is a process that is involved in viral (influenza) clearance [76]. A study performed in Wuhan, China investigated the relationship between gut microflora composition and the predisposition of healthy individuals to SARS-CoV-2 infection [77]. Gou and colleagues (2020) showed that the genus *Bacteroides* and *Streptococcus*, as well as the order Clostridiales, had a negative correlation with the majority of the tested cytokines (IL-1β, IL-2, IL-4, IL-6, IL-8, IL-10, IL-12p70, IL-13, TNF-α, and IFN-γ), while the genus *Lactobacillus*, *Ruminococcus*, and *Blautia* displayed positive associations with the referred cytokines [77]. Specifically, individuals with increased numbers of *Lactobacillus* had higher levels of IL-10, an anti-inflammatory cytokine [77]. In contrast, individuals displaying higher *Ruminococcus gnavus* showed increased levels of pro-inflammatory cytokines as well as more pronounced disease severity [77]. Thus, it is important to elucidate the role of gut microbiota and the gut–lung axis in respiratory diseases in order to uncover their therapeutic role in the treatment of COVID-19.

**Figure 3:**
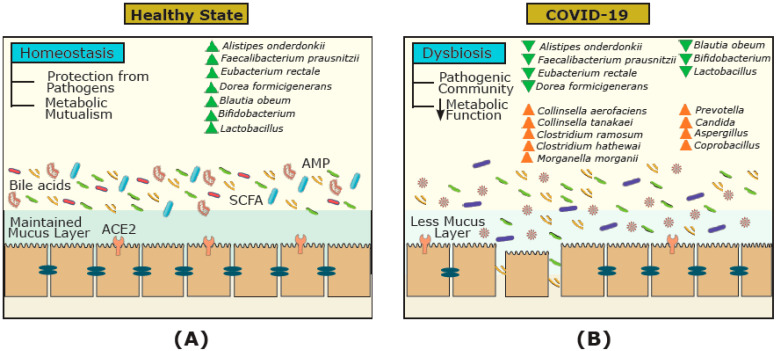
The gut microbial ecosystem and underlying mechanisms involved in COVID-19 infection. The gut microbiota of (**A**) healthy individuals, and (**B**) COVID-19-infected individuals. In healthy individuals, the gut harbors diverse communities and metabolites (e.g., short chain fatty acids (SCFAs) and bile acids) that vary from the gut microbial communities of COVID-19-infected individuals. During COVID-19, the dysbiosis of the gut microbial ecosystem, the reduction in metabolites involved, and downregulation of angiotensin-converting enzyme 2 (ACE2) receptors can affect the immune system (e.g., reduction in antimicrobial peptides (AMPs)) and worsen conditions.

Conversely, viral respiratory infections such as COVID-19 can affect the gut microbiota and lead to bacterial infections requiring antibiotic treatment, which can also increase the risk of rCDI and the need for FMT [78,79,80]. COVID-19 may affect the gut via ACE2 receptors, which can regulate intestinal amino acid homeostasis, the expression of AMPs, inflammation in the gut, and the ecology of the gut microbiome [81]. During SARS-CoV-2 infection, ACE2 expression is downregulated, leading to gut microbiome dysbiosis and disrupting the metabolic homeostasis, and altering the level of intestinal metabolites such as amino acids, bile acids, and SCFAs [29]. SARS-CoV-2, for example, has been shown to decrease the SCFA butyrate [82], which is a key modulator of the immune system in the intestinal tract [83] and the lungs [84]. Whether these changes can affect the gut mucosal integrity, triggering inflammation, and cytokine release remain to be explored. Furthermore, increased *Prevotella* and decreased *Lactobacillus* and *Bifidobacterium* have been observed in clinical samples of SARS-CoV-2-infected patients [85,86,87]. Increased *Prevotella* in the gut, for example, can mediate inflammatory response via toll-like receptor 2 (TLR2) activation, which can lead to inflammation and T-helper cell 17 (Th17) immune response [69]. Thus, it is reasonable to postulate that the expression of ACE2 receptors, the production of gut microbial metabolites (i.e., SCFAs and bile acids), and the immune system can be affected by COVID-19, and significantly differ in COVID-19-infected individuals compared to healthy non-infected individuals (Figure 3). However, how long such changes persist and if all these changes are reversible are not known.

### 3.1. A Donor Perspective

Current guidelines for FMT include the utilization of stool donated pre-COVID-19 pandemic until proper testing and screening protocols are available [30], which can be burdensome for stool banks and donors. During COVID-19 and post-COVID-19 pandemic, practitioners using FMT treatment may be faced with three possibilities, including donor samples from (i) healthy non-infected donors (Figure 1A,B); (ii) recovered-donors after COVID-19 infection (Figure 1A,B); and (iii) asymptomatic COVID-19 donors (Figure 1A). After stringent screening protocols of healthy non-infected donors, samples can be accepted for FMT (group a). In contrast, samples from COVID-19-infected individuals must be excluded, because COVID-19 may be transmitted through FMT (group c) [26]. In addition, previously donated stool, up to four weeks before the occurrence of symptoms/COVID-19 diagnosis, should be discarded because evidence suggests that SARS-CoV-2 is able to remain in stool up to four weeks after infection [88]. However, the inclusion of previously healthy donors who have recovered from COVID-19 (group b) is a complex matter. Previous studies have proposed that a COVID-19-infected donor must be excluded, but can be re-tested in 30 days and included if they have tested negative for COVID-19 in their stool sample and are symptom-free [33]. However, because COVID-19 can affect the gut–lung axis, previously healthy donors who have recovered (group b) may continue to have disrupted gut microbiota and metabolites essential for successful FMT treatment outcomes. For example, it has been suggested that an ideal donor should have high *Lachnospiraceae*, *Ruminococcaceae*, and *Clostridium scindens* which are positively associated with secondary bile acids that inhibit CDI germination [89,90,91]. Moreover, FMT restores SCFAs metabolism, with immune modulatory effects in rCDI patients [92]. However, it is important to note that donors who have recovered from COVID-19 may have an altered gut microbiota and are missing key microbiota leading to essential functional groups such as bile acids and SCFAs. Thus, COVID-19 infection may reduce the efficacy of FMT in clearing *C. difficile* infection. In a study by Liu and colleagues (2021), FMT was conducted on 11 discharged COVID-19 patients in order to investigate the potential beneficial effects of FMT on the gut microbiota and the immune system [93]. Post-FMT, five participants reported the alleviation in gastrointestinal symptoms. Although microbial richness increased, the overall diversity did not differ post-FMT [93]. Specifically, *Bifodobacterium* and *Faecalibacterium* significantly increased post-FMT; therefore, FMT may serve as a potential therapeutic intervention for patients who continue to experience gastrointestinal symptoms following COVID-19.

Beyond the gut bacterium, studies have also examined the role of the gut mycobiome and virome on FMT efficacy. For example, Zuo and colleagues found a negative relationship between the abundance of fungi such as *Candida albicans* in donor stool and FMT efficacy [41]. The reduction in the abundance of *Caudovirales* bacteriophages and an increase in *Microviridae* abundance; specifically, a higher abundance of *Eel River basin pequenovirus* as a potential Proteobacteria predator, were shown to be related to FMT efficacy in CDI patients. Thus, in order to uncover mechanisms involved in FMT efficacy during and post-COVID-19, it is fundamental to include the relative contribution of all domains.

Demographic factors such as age, sex, and ethnicity may also need to be taken into consideration with regard to donor selection in the COVID-19 era. To the best of our knowledge, no one has suggested that the sex of the donor should be considered during FMT screening protocols. However, FMT stool banks may face a reduction in the numbers of male donors, because men might be at a higher risk for severe illness and fatal outcomes from COVID-19 [73,94]. Contemporary reports indicate that circulating levels of ACE2 are higher in men compared to women, while others found no sex differences, but reported higher ACE2 in older women [95,96]. Beyond environmental and social differences between men and women (e.g., lifestyle, social stresses, access to healthcare, smoking, etc.) that can contribute to disease predisposition, sex chromosomes and sex-based immunological patterns can also contribute to COVID-19 pathogenesis [97]. For example, antiviral immunity differs between the sexes [98], with the number and activity of innate immune cells, including monocytes, macrophages, and dendritic cells, as well as inflammatory immune responses being higher in females [99,100]. For example, females have an increased expression of TLR7 levels [101,102], while males have lower CD3+ and CD4+ cell counts and helper T cell type 1 (Th1) responses [103,104]. Sex-differences are also associated with the overall gut microbiota structure, contributing to COVID-19 pathogenesis [105,106]. For example, in a study by Tekagi and colleagues, significant increases in genera *Prevotella*, *Megamonas*, *Fusobacterium*, and *Megasphaera* in males, and *Bifidiobacterium*, *Ruminococcus*, and *Akkermansia* in females were identified [106]. However, males and females did not differ significantly in their microbial diversity [106]. Studies based on sex differences in gut microbial composition and COVID-19 development are still rare and require further investigation.

Stool banks may also face a reduction in the number of donors from African Americans due to higher COVID-19 mortality among African Americans than in White Americans [107]. For example, chronic conditions, especially diabetes, are common in African and South Asian minority groups [108], which contribute to worse outcomes of COVID-19. Certain minority groups may also have a higher risk for COVID-19 due to lifestyle, environmental factors, and socioeconomic factors such as lower education, higher poverty, higher uninsured rates, and decreased access to healthcare [109,110]. In addition, ethnicity and dietary differences are associated with variations in microbial composition and abundances, more strongly than other factors such as genetics, age, sex, and body mass index [111]. Studies based on demographic-related differences in gut microbial composition and COVID-19 development are still insufficient and require further investigation.

### 3.2. A Patient Perspective

A critical consideration for FMT efficacy and durability is that the microbial consortium of the donors is not the only key player. It has been shown that the idea of “super donors” is oversimplified and that a trans-kingdom battle exists between the donor and recipient of FMT treatment [112]. The existing endogenous microbiome in recipients and their functions can also play a significant role in determining the colonization of those exogenous species. For example, focusing on bacterial engraftment, Smillie and colleagues suggested that selective forces in the patient’s gut (host control), rather than input dose dependence (bacterial abundance in the donor and patient), can determine bacterial abundance after FMT and, subsequently, its efficacy [113]. Thus, FMT recipients should be screened for COVID-19 symptoms and exposure history. Figure 1 shows two FMT scenarios during and post-COVID-19 infection where the FMT recipient has tested positive for COVID-19 infection, which may potentially affect FMT efficacy in clearing *C. difficile* infection (Figure 1A,B). If possible, FMT treatment should be delayed if recipients are experiencing active COVID-19 symptoms, because COVID-19 may affect the lung microbiota, gut microbiota, and their associated metabolites (e.g., bile acids, SCFAs, etc.), which may affect FMT efficacy. For patients who have recovered from COVID-19 (Figure 1B), FMT may not be as effective because the gut ecosystem and the immune response of the rCDI patient following COVID-19 may differ from rCDI individuals without COVID-19, and potentially interfere with the successful engraftment of donor microbiota, reducing FMT success.

The initial weeks following FMT are critical in breaking the cycle of CDI recurrence, because most relapses occur during the first 2–4 weeks after treatment [114,115]. Therefore, changes during this window of time are critical for mechanistic investigations of rCDI therapies during COVID-19 and post-COVID-19 pandemic. In addition, the elderly population and immunocompromised individuals are at high risk for COVID-19, as well as suffering from an increased incidence and adverse outcomes when developing rCDI [94,116,117,118,119]. It is also well known that gut microbial diversity and the abundance of genes involved in SCFA production decrease with age, while comorbidities may increase [120]. Aging can also affect the immune system, with systemic inflammation being one of the hallmarks of aging [121], which in turn yields susceptibility to COVID-19 and rCDI.

## 4. Future Outlook

Here, we highlighted the urgent need to develop comprehensive and optimized screening protocols for stool donors in order to ensure the safety and efficacy of FMT during COVID-19 and post-COVID-19 pandemic. Under this mandate, donors as well as recipients’ perspectives must be considered. The interplay between gut microbial composition and function with COVID-19 development, along with demographic factors such as sex, age, and ethnicity, illustrate the complex host factors involved in health and disease states. While the race to vaccinate the world continues, uncovering the bidirectional effects of COVID-19 and the gut–lung axis is crucial for tailoring conventional FMT strategies to remain safe and effective during and after the pandemic. Moreover, elucidating the role of gut microbiota and the gut–lung cross-talk in respiratory diseases can lead to novel microbiome-based preventative and therapeutic interventions for COVID-19 and other pandemics in the future.

## Data Availability

Not applicative.
